# Working around the Clock: Is a Person’s Endogenous Circadian Timing for Optimal Neurobehavioral Functioning Inherently Task-Dependent?

**DOI:** 10.3390/clockssleep4010005

**Published:** 2022-02-11

**Authors:** Rachael A. Muck, Amanda N. Hudson, Kimberly A. Honn, Shobhan Gaddameedhi, Hans P. A. Van Dongen

**Affiliations:** 1 Sleep and Performance Research Center, Washington State University, Spokane, WA 99202, USA; rachael.muck@wsu.edu (R.A.M.); amanda.hudson@wsu.edu (A.N.H.); kimberly.honn@wsu.edu (K.A.H.); 2 Department of Translational Medicine and Physiology, Elson S. Floyd College of Medicine, Washington State University, Spokane, WA 99202, USA; 3 Department of Biological Sciences and Toxicology Program, North Carolina State University, Raleigh, NC 27695, USA; sgaddam4@ncsu.edu; 4 Center for Human Health and the Environment, North Carolina State University, Raleigh, NC 27695, USA

**Keywords:** cognitive throughput, constant routine, inter-individual differences, simulated shift-work, sleep/wake homeostasis, subjective sleepiness, vigilant attention

## Abstract

Neurobehavioral task performance is modulated by the circadian and homeostatic processes of sleep/wake regulation. Biomathematical modeling of the temporal dynamics of these processes and their interaction allows for prospective prediction of performance impairment in shift-workers and provides a basis for fatigue risk management in 24/7 operations. It has been reported, however, that the impact of the circadian rhythm—and in particular its timing—is inherently task-dependent, which would have profound implications for our understanding of the temporal dynamics of neurobehavioral functioning and the accuracy of biomathematical model predictions. We investigated this issue in a laboratory study designed to unambiguously dissociate the influences of the circadian and homeostatic processes on neurobehavioral performance, as measured during a constant routine protocol preceded by three days on either a simulated night shift or a simulated day shift schedule. Neurobehavioral functions were measured every 2 h using three functionally distinct assays: a digit symbol substitution test, a psychomotor vigilance test, and the Karolinska Sleepiness Scale. After dissociating the circadian and homeostatic influences and accounting for inter-individual variability, peak circadian performance occurred in the late biological afternoon (in the “wake maintenance zone”) for all three neurobehavioral assays. Our results are incongruent with the idea of inherent task-dependent differences in the endogenous circadian impact on performance. Rather, our results suggest that neurobehavioral functions are under top-down circadian control, consistent with the way they are accounted for in extant biomathematical models.

## 1. Introduction

Neurobehavioral functioning is strongly influenced by the two processes of sleep/wake regulation: a homeostatic process (“Process S”), which degrades neurobehavioral functioning over time awake and restores it during sleep; and a circadian process (“Process C”), which cycles between promoting and depressing neurobehavioral functioning across time of day [1,2]. The combined effect of these two processes governs changes in waking neurobehavioral functioning over time, such that day shift-workers tend to exhibit stable, near-optimal performance through the workday [3,4], whereas night shift-workers typically experience a steady decline of performance through the night [5,6]. Inter-individual differences in the contributions of the two processes notwithstanding [7,8], these dynamics are well understood [9,10] and have led to the development of biomathematical models predicting neurobehavioral performance across a wide range of sleep/wake/work scenarios [11,12]. Such biomathematical models can be used to prospectively predict performance impairment in shift-workers [13,14] and may be implemented in 24/7 operations as a tool for fatigue risk management [15,16].

The importance of circadian rhythms for neurobehavioral performance has long been recognized [17,18,19]. It has been observed, however, that the circadian rhythmicity of neurobehavioral functioning may be task-dependent. Early studies reported that under conditions of sleep deprivation and forced desynchrony, the timing of optimal functioning varies across tasks [20,21]. For example, performance on complex cognitive tasks (e.g., verbal reasoning) was found to peak in the late morning [22,23], whereas simple perceptual-motor task performance was optimal in the late afternoon [24]. These differences suggested the involvement of multiple, distinct underlying brain oscillators [25,26] and led researchers to conclude that “it is as incorrect to speak of a single performance rhythm as it is to speak of single physiological rhythm” [26] (p. 544). Furthermore, functional magnetic resonance imaging (fMRI) data from a recent sleep deprivation study, captured during a sustained attention test administered multiple times over the circadian cycle, showed variation in the phase of circadian rhythmicity between brain regions. According to the investigators, this “rules out a global task-independent circadian influence and suggests the influence of a local, region-specific, task-dependent circadian signal” [27] (p. 690). However, when performance was investigated under constant routine (CR) conditions, differences in the circadian rhythms of performance between short-term memory and calculation performance tasks and subjective alertness [28] as well as between serial search, verbal reasoning, manual dexterity, and visual vigilance tasks [29] were largely absent. As Monk and colleagues observed, their CR study “confirmed the suggestion made by Johnson et al. (1992) that inter-task differences, which may result in differences in time of peak and trough under a normal nychthemeral routine, can fail to do so when the sleep/wake cycle is suspended, and endogenous circadian rhythms are ‘unmasked’ using a constant conditions protocol” [29] (p. 14). These strictly controlled laboratory studies challenged the concept of task-dependent circadian rhythms in neurobehavioral functioning; yet, subsequent publications on the topic have led to renewed interest in the idea [30,31,32,33].

In studies investigating task-dependent variation in the timing of circadian rhythmicity in neurobehavioral functioning, the influence of the circadian process (time of day) needs to be dissociated from the influence of the homeostatic process (time awake) [34]. Because during periods of wakefulness, when neurobehavioral functioning is measured, time of day and time awake change hand in hand, disentangling the circadian (rhythmic) and homeostatic (trending) processes involved must be done post hoc. This is mathematically and statistically complicated and prone to misestimation of rhythm parameters [35]. The problem is compounded by the task-dependence of vulnerability to neurobehavioral impairment due to sleep loss [36,37], which may be mediated by use-dependent, pathway-specific degradation of neuronal processing [38] and implies that there are task-specific differences in the impact of the homeostatic process [39]. These issues can be overcome by explicitly accounting for inter-individual variability during data analysis [7] but, to date, that approach has not been applied to the evaluation of putative task differences tied to circadian rhythmicity. Various other potential confounds further cast doubt on the reliability of reports of task-specificity in the timing of circadian rhythms [34]. That is, different performance tasks are differentially susceptible to phenomena that can mask circadian rhythmicity, including practice effects [40], performance strategy changes [41], and temporal changes in speed/accuracy trade-offs [42]. These masking effects could create the appearance of task-dependent differences in the effect of circadian rhythmicity on neurobehavioral functioning even when no such differences are endogenously produced.

Here, we addressed these issues by means of a simulated shift-work protocol with randomization to either a day shift condition or a 12 h offset night shift condition, followed by a CR protocol, during a 7-day/6-night laboratory study. The CR protocol served to measure neurobehavioral functioning, at 2 h intervals, under strictly controlled conditions, and to expose the endogenous circadian rhythmicity [43]. The neurobehavioral assays we used were functionally distinct [36,40], suitable for repeated administration, and not subject to masking from practice, strategy changes, or dynamically shifting speed/accuracy trade-offs. The test battery included a psychomotor vigilance test (PVT) [44], a computerized digit symbol substitution test (DSST) [45], and the Karolinska Sleepiness Scale (KSS) [46]. The two conditions of the simulated shift-work protocol that preceded the CR protocol maximized variability in circadian alignment and optimized the mathematical/statistical disentanglement of the effects of the circadian and homeostatic processes on task performance. With mixed-effects regression methodology developed specifically to account for differences between individuals [47], we investigated whether the effect of the circadian process on neurobehavioral functioning is, in fact, fundamentally task-dependent.

## 2. Materials and Methods

### 2.1. Subjects

Fourteen healthy adults (10 men and 4 women), ranging in age from 22 to 34 years (mean ± SD: 25.8 ± 3.2 years), participated in a highly controlled laboratory study. They were physically and psychologically healthy as determined by physical exam, history, and questionnaires. Subjects were normal sleepers with no sleep or circadian disorders as verified with questionnaires and baseline polysomnography, were neither extreme morning types nor extreme evening types as assessed by questionnaire [48], reported no shift-work in the prior three months, and did not travel across time zones during the month prior to participation. They reported habitual sleep durations between 6 h and 10 h per night and wake-up times between 06:00 and 09:00.

During the seven days before the laboratory study, subjects maintained their habitual sleep/wake schedule, without napping, as verified with wrist actigraphy, sleep diaries, and call-ins of bedtimes and waketimes on a time-stamped voice recorder. During the seven days prior and while in the laboratory, subjects refrained from caffeine and alcohol intake, smoking, and drug use (except oral contraceptives), as verified with breathalyzer and urine tests. The study was approved by the Institutional Review Board of Washington State University. Subjects gave written, informed consent and were paid for their time.

### 2.2. Experimental Design

**Figure 1:**
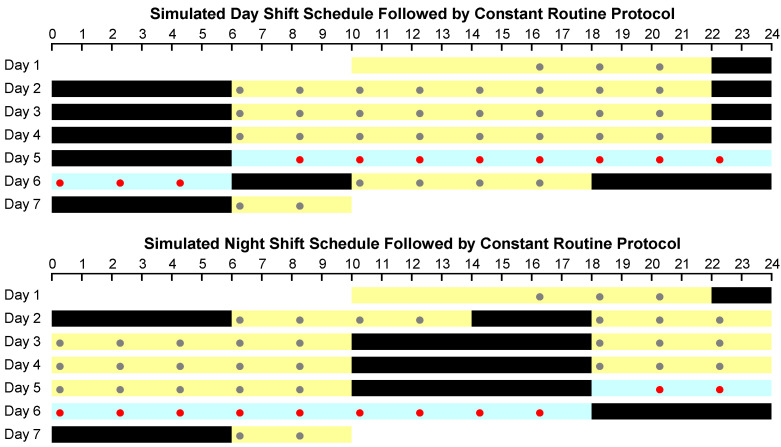
Schematic of the study design, showing the simulated day shift condition (**top**) and the simulated night shift condition (**bottom**). In each panel, time of day progresses from left to right, and days progress from top to bottom. Black bars indicate sleep opportunities; yellow bars indicate scheduled wakefulness. The 24 h waking period of the constant routine (CR) protocol that followed three days of simulated day or night shift schedule is shown in blue. Dots denote neurobehavioral test bouts; red dots indicate the test bouts used for analysis.

Subjects completed a 7-day/6-night in-laboratory study, which was designed as shown in Figure 1. Following a baseline day and night (8 h sleep opportunity: 22:00–06:00), subjects were randomized to one of two conditions (seven subjects each): three days of a simulated day shift schedule with 8 h nighttime sleep opportunities (22:00–06:00); or a daytime nap (14:00–18:00) followed by three days of a simulated night shift schedule with 8 h daytime sleep opportunities (10:00–18:00). In both conditions, light exposure during wakefulness was low (<50 lux). Immediately after the last sleep opportunity of the three-day simulated day or night shift schedule, subjects underwent a 24 h CR protocol, during which they stayed awake under continuous behavioral monitoring, ate a small isocaloric snack every hour, and maintained a semi-recumbent posture under constant dim light (<50 lux) and fixed ambient temperature (21 ± 1 °C). The study ended with a recovery period, after which subjects left the laboratory.

Sleep periods were recorded polysomnographically and scored visually using standard criteria promulgated by the American Academy of Sleep Medicine [49]. Sleep findings for this study have been documented previously [50]; here, the sleep records were used to assess the timing of final awakening before the start of the CR protocol (mean ± SD for the day shift condition: 05:59 ± 1 min, for the night shift condition: 17:53 ± 16 min).

During the CR protocol, blood was collected at intervals of 1–3 h through an intravenous catheter. Blood samples were used to measure circulating melatonin (radioimmunoassay IB88111, IBL), and the dim light melatonin onset (DLMO) was assessed as a marker of the timing of the endogenous circadian pacemaker as described previously [51]. For one subject in the simulated night shift condition, the subject-specific DLMO could not be assessed reliably, because the individual’s melatonin concentration did not reach the 10 pg/ml threshold used to define DLMO [51]. As our statistical approach involved using subject-specific circadian time based on DLMO (see below), this subject was removed from the data set as a whole, leaving data from a total of thirteen subjects (*n* = 7 in the day shift condition and *n* = 6 in the night shift condition) for analyses.

### 2.3. Neurobehavioral Assays

Throughout periods of wakefulness during the study, including the CR protocol, subjects completed three neurobehavioral assays every 2 h (Figure 1): the KSS, DSST, and PVT. The KSS was administered both before and after the DSST, which was followed by the PVT.

The KSS is a self-report scale of subjective sleepiness [52] with response options ranging from 1 (extremely alert) to 9 (very sleepy, fighting to stay awake). Scores on the two KSS administrations in each test bout were averaged to yield one KSS outcome measure. The KSS is highly sensitive to circadian rhythmicity [46] and functionally distinct from the DSST and PVT both within [45] and between [36] individuals.

The DSST requires subjects to match digit and symbol stimulus pairs as quickly as they can while prioritizing accuracy [53]. In the 3 min computerized version used here [45], a key is displayed at the top of the screen, indicating a series of different symbols (e.g., star, plus sign) paired with the digits 1–9. In the center of the screen, a symbol is presented, and the subject must type the corresponding digit using the number keys at the top of the keyboard. After each response, the symbol in the center is replaced with a new one; subjects determine the pacing of the task by the speed of their responses. The key remains the same throughout the task duration, but the pairings vary between test bouts. Cognitive throughput (number of correct responses) was used as the outcome measure of interest. The DSST is highly sensitive to circadian rhythmicity [34]. The task shows a minor but persistent practice effect [45]; however, at the start of the CR protocol the practice effect would have approached asymptote as, by that time, subjects had already performed the task more than two dozen times across the simulated shift work days. The components of cognition involved in the DSST are partially distinct from those involved in the PVT [40], and DSST performance has been shown to be functionally distinct from PVT performance both within [45] and between [36] individuals.

The PVT is a 10 min computer-paced, one-choice reaction time task requiring subjects to respond as fast as possible to a stimulus presented as a rolling millisecond counter that appears in random 2–10 s intervals [54]. Rather than the conventionally used number of lapses of attention [55], which exhibits a floor effect, an index of the fidelity of information processing (log-transformed signal-to-noise ratio; LSNR) was used as the outcome measure of interest [56]. The PVT does not show any meaningful practice effects [45], but is highly sensitive to circadian rhythmicity [34].

To account for any idiosyncratic differences between subjects in basal neurobehavioral functioning, outcome measures were expressed relative to the baseline average, calculated using the second through fourth test bouts on day two (at 08:15, 09:20, and 12:00), leaving enough time after the first in-laboratory sleep opportunity to dissipate sleep inertia [57] and preceding the daytime nap at the start of the simulated night shift condition. Difference scores for the DSST were inverted so that larger values corresponded to greater neurobehavioral impairment for all three assays.

### 2.4. Statistical Methods

**Figure 2:**
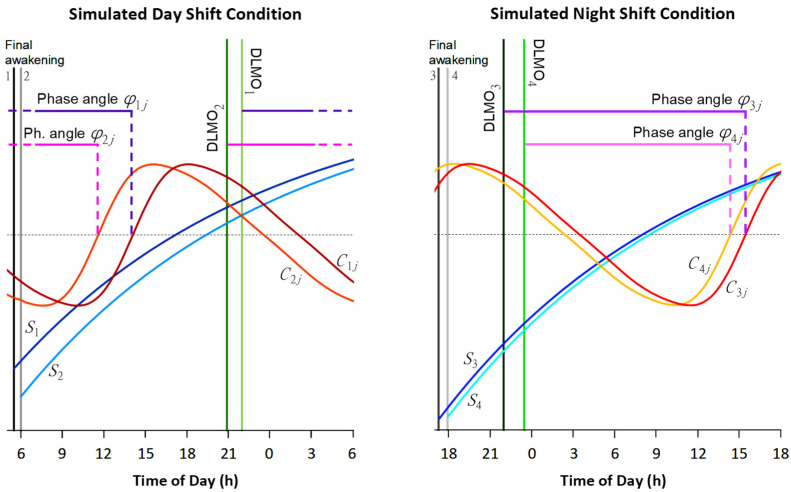
Schematic of the statistical approach. We modeled the effects of the circadian process *C* (orange-red curves) and the homeostatic process *S* (blue curves) on neurobehavioral functioning, illustrated here with four example (hypothetical) individuals (arbitrarily numbered 1–4) for a given neurobehavioral task *j*, plotted against clock time over the 24 h period of wakefulness of the CR protocol that followed the simulated day shift condition (**left panel**) and the simulated night shift condition (**right panel**). The statistical model included task-specific scaling factors for processes *C* and *S* and a task-specific intercept (not shown). We accounted for individual differences by expressing *C* in subject-specific circadian time, relative to individual DLMO (green vertical lines), and *S* in subject-specific time awake, relative to individual time of final awakening (gray-black vertical lines), and by estimating individualized initial state for process *S* at final awakening. Importantly, we also estimated individualized phase angles *φ_ij_* (purple-pink horizontal lines), denoting subject-specific circadian timing of the upward zero crossing (intersection with dotted horizontal line) of the effect of process *C* on performance for neurobehavioral task *j*. We compared the means of these phase angles over all subjects between our three distinct neurobehavioral tasks to address the question whether the endogenous circadian timing for optimal functioning was inherently task-dependent.

Our statistical approach is illustrated in Figure 2. It is based on the equations for the two-process model [58] and our earlier work on the estimation of the contributions of the homeostatic and circadian processes to neurobehavioral impairment during continuous wakefulness [7]. As was shown, the temporal dynamics of performance *y_j_* across a period of wakefulness for a given task *j* can be described with the following regression model:

(1) $$y_{j}(t_{a}^{},t_{c})=\beta_{j}S(t_{a})-\gamma_{j}C(t_{c})+\kappa_{j}+\varepsilon_{j}(t_{c}),$$

where *S* and *C* represent the homeostatic and circadian processes, respectively; *β_j_* and *γ_j_* are task-specific scaling factors for processes *S* and *C*, respectively; *κ_j_* is a task-specific intercept; *t*_a_ denotes time awake (waking time since final awakening immediately before the start of the CR protocol); *t*_c_ denotes circadian time (time of day expressed relative to DLMO); and *ε_j_* is residual variance assumed to be normally distributed over time with zero mean and task-specific variance *σ_j_*^2^.

For a given individual *i*, the two-process model equation for the homeostatic process *S* during wakefulness [58] can be shown to be written as [7]:

(2) $$S_{i}(t_{a,i})=(S_{0,i}-1)e^{-t_{a,i}/\tau_{w}}+1,$$

where *τ*_w_ = 18.2 h is the previously estimated time constant of homeostatic build-up during wakefulness [58]; *t*_a,*i*_ is the subject-specific time awake (depending on the timing of the individual’s final awakening); and *S*_0,*i*_ is the initial subject-specific state of the homeostatic process at the start of the waking period. Note that because our study had a between-subjects design with different individuals assigned to the day versus night shift condition, *S*_0,*i*_ is automatically condition-specific (and thereby accounts for any sleep-related differences between conditions). Taking the homeostatic process and the intercept in the model together, we can write:

(3) $$\beta_{j}S(t_{a,i})+\kappa_{j}=\beta_{j}((S_{0,i}-1)e^{-t_{a,i}/\tau_{w}}+1)+\kappa_{j}=B_{j}e^{H_{i}}e^{-t_{a,i}/\tau_{w}}+K_{j},$$

where *B_j_* is a task-specific scaling factor, *H_i_* is a subject-specific scaling coefficient thereof, and *K_j_* is a task-specific intercept.

Further, the subject-specific and (potentially) task-dependent endogenous circadian process *C* is given by [58]:

(4) $$C_{ij}(t_{c,i})=A\sum_{m=1}^{5}a_{m}\sin(2m\pi (t_{c,i}-\phi_{ij})/24),$$

where the *A* and *a_m_* coefficients are previously assessed constants [58]; *t*_c,*i*_ is the subject-specific circadian time (depending on the timing of the individual’s DLMO); and *φ_ij_* is a subject-specific and (potentially) task-dependent phase angle (i.e., circadian timing of the upward zero crossing of process *C* relative to the subject-specific DLMO). Note again that because our study had a between-subjects design, *φ_ij_* is automatically condition-specific.

Putting these pieces together, the temporal dynamics of performance *y_ij_* across a period of wakefulness for a given individual *i* and given task *j* can be modeled as follows:

(5) $$y_{ij}(t_{a,i}^{},t_{c,i})=B_{j}e^{H_{i}}e^{-t_{a,i}/\tau_{w}}-G_{j}\sum_{m=1}^{5}a_{m}\sin(2m\pi (t_{c,i}-\phi_{ij})/24)+K_{j}+\varepsilon_{ij}(t_{c,i}),$$

where *G_j_* is a task-specific scaling factor for the circadian process; and *ε_ij_* is error variance assumed to be normally distributed over subjects and time with zero mean and task-specific variance *σ_j_*^2^. For the *k* = 3 different neurobehavioral assays considered in our study, this regression equation has *k* + 1 = 4 subject-specific parameters, namely *φ_ij_* (*j* = 1,…, *k*) and *H_i_*. These can be modeled by independent, normally distributed random effects with parameter-specific mean *Φ_j_* and zero and parameter-specific variance *ω_j_*^2^ and *ϖ*^2^, respectively. (Note that the normal distribution of *H_i_* makes the overall scaling of the homeostatic process log-normally distributed over subjects.)

To allow for direct comparisons between neurobehavioral assays within the same regression model, we normalized the data *y_ij_* for each of the assays using the task-specific grand mean and standard deviation. We then fitted the model to the overall data set of the three assays combined using non-linear mixed-effects regression [47] implemented in SAS 9.4 (SAS Institute Inc., Cary, NC, USA). Contrasts were included to compare parameter estimates among the assays, with the comparison between the mean phase angles *Φ_j_* being of primary interest.

## 3. Results

Following the three days of simulated night shift, compared to simulated day shift, there was a modest delay of 87 min (±45 min SE; *t*_11_ = 1.92, *p* = 0.082) in the circadian process as estimated by the timing of the DLMO, which occurred at 23:01 (±95 min SD) in the night shift condition versus 21:34 (±68 min SD) in the day shift condition. Our DLMO data are consistent with natural variability in circadian phase [59,60] and prior observations of limited circadian phase shifting in night shift protocols under dim light conditions [61]. Because the homeostatic process is a function of time awake and not time of day (and vice versa for the circadian process), it follows that the simulated night shift condition produced an average shift of 10.6 h in the alignment of the homeostatic process relative to the circadian process during the subsequent 24 h CR protocol. This considerable dispersion confirmed the effectiveness of the study protocol and helped to optimize the statistical analysis and parameter estimation of the circadian process. To verify that the statistical model of Equation (5) was well-parameterized for investigating endogenous circadian rhythmicity, we checked the parameter correlation matrix and made sure all estimated parameters (other than the intrinsically correlated homeostatic parameters *B_j_* and *K_j_*) had low pairwise correlations. They were between −0.37 and 0.36, confirming excellent parameter estimability.

**Figure 3:**
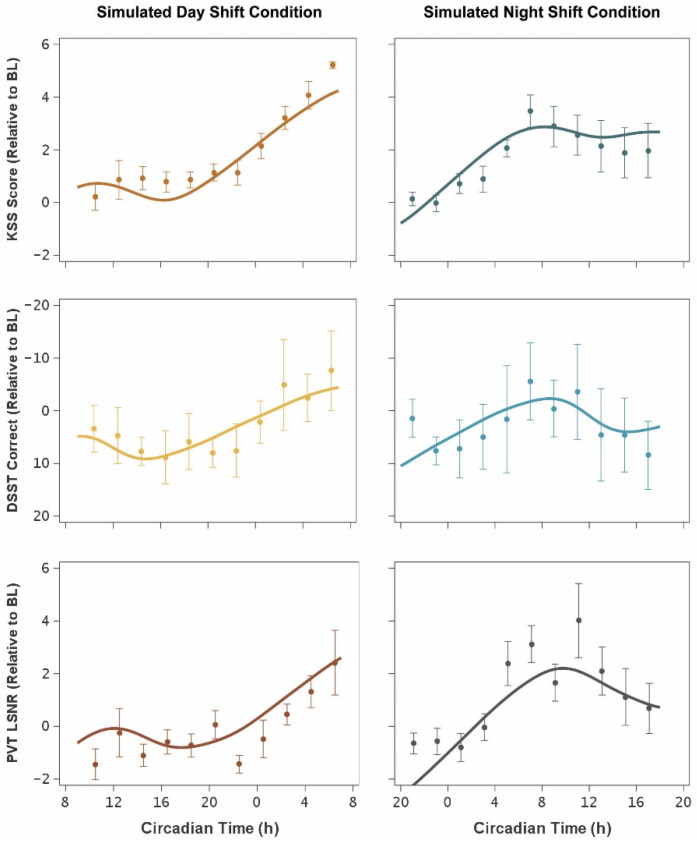
Temporal profiles of neurobehavioral functioning during the 24 h CR protocol. The graphs show means (±SE) for KSS (**top**), DSST (**middle**), and PVT (**bottom**), expressed relative to baseline (BL) and plotted against mean circadian time (i.e., relative to DLMO), for the simulated day shift condition (left) and the simulated night shift condition (right). Curves represent the mean (accounting for inter-individual differences) of the fitted statistical model of Equation (5). Upwards indicates worse neurobehavioral functioning in all panels.

Figure 3 shows the temporal profiles of the three neurobehavioral outcomes as observed during the 24 h CR protocol following the simulated day and night shift protocols (left and right panels, respectively), plotted against circadian time (i.e., relative to DLMO). The statistical model curves in the day shift condition (left panels) show the expected profile of low neurobehavioral impairment during the first ~16 h of wakefulness constituting the biological day, followed by a steady increase of impairment into the biological night [3]. Likewise, the statistical model curves in the night shift condition (right panels) show the expected profile of steadily increasing impairment through the biological night and into the subsequent biological day, followed by a partial (circadian-mediated) rebound toward the end of the biological day [4].

**Figure 4:**
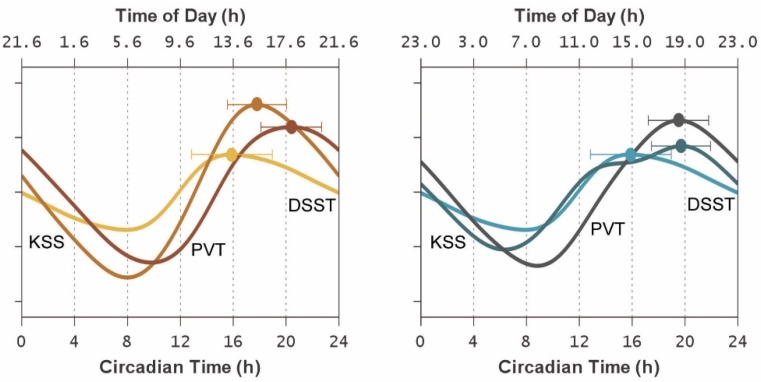
Endogenous circadian rhythm contributions to neurobehavioral functioning during the 24 h CR protocol. The graphs show the mean (accounting for inter-individual differences) of the circadian component of the fitted statistical model of Equation (5), plotted against circadian time (i.e., relative to DLMO) on the bottom axes and against corresponding mean clock time on the top axes. Curves represent the endogenous circadian rhythm influence on each of the three neurobehavioral outcomes after normalization (i.e., as *z* scores, to make them comparable in vertical scale) for the simulated day shift condition (**left**) and the simulated night shift condition (**right**) using the same color scheme as in Figure 3. Upwards indicates greater circadian drive (corresponding with better neurobehavioral functioning). The secondary bump in the KSS curve in the simulated night shift condition (around 14 h after DLMO) is a result of convolution of the subject-specific circadian rhythm contributions encompassed in the mean. Dots and whiskers represent the task-specific circadian peaks and their 95% confidence intervals. Note the relatively tight clustering of the peaks, regardless of condition, in the late biological afternoon (approximately 16–20 h after DLMO).

Figure 4 shows the endogenous circadian rhythm contribution to the temporal profiles of each of the three neurobehavioral outcomes during the 24 h CR protocol following the simulated day and night shift conditions, as estimated using Equation (4) as embedded in the statistical model of Equation (5). The magnitude of the contribution of the circadian process, to be interpreted relative to the homeostatic process, was smaller for the DSST than for the KSS and PVT (*F*_2,9_ = 5.61, *p* = 0.026). However, even though the statistical model was fit with task-specific and condition-specific parameters and also accounted for inter-individual differences, the timing of the effect of the circadian process on neurobehavioral functioning, relative to subject-specific DLMO, was consistent between the day and night shift conditions for all three neurobehavioral functioning outcomes. This was corroborated by comparisons of the subject-specific *φ_ij_* estimates, i.e., the empirical Bayes estimates (EBEs) for the task-specific random effects on the circadian phase angles relative to subject-specific DLMO in the statistical model of Equation (5). There were no significant differences between conditions in the EBEs for the KSS (*F*_1,11_ = 0.01, *p* = 0.90), DSST (*F*_1,11_ = 0.83, *p* = 0.38), and PVT (*F*_1,11_ = 0.37, *p* = 0.56), indicating no systematic shifting between conditions in the timing of the circadian contribution, relative to DLMO, to neurobehavioral functioning. 

As such, the peak times of the endogenous circadian contribution to neurobehavioral functioning varied little between the neurobehavioral outcomes, and there was substantial overlap among the 95% confidence intervals (Figure 4). Notably, there was no significant effect of task on the timing of the circadian peaks relative to DLMO (*F*_2,9_ = 1.45, *p* = 0.28).

## 4. Discussion

This study provides proof of principle for our approach to investigating whether the circadian rhythmicity of neurobehavioral functioning is task-dependent, addressing limitations of earlier research on this topic. In contrast with previous reports that circadian rhythms peak in the morning or afternoon depending on the task at hand [21,26,30], we found no evidence of a task-based dissociation in optimal circadian timing for three functionally distinct neurobehavioral outcomes. During a 24 h CR protocol, preceded by either a three-day simulated day shift schedule or a three-day simulated night shift schedule, circadian peak times clustered approximately 16–20 h after DLMO—consistent with the height of endogenous circadian drive for alert wakefulness during the “wake maintenance zone” [62] in the late afternoon [63,64,65]. Differences in circadian peak times between the neurobehavioral functions we measured were within the margin of error and no greater than our 2 h sampling interval (Figure 4). Thus, we found no evidence that the circadian timing for optimal neurobehavioral functioning is inherently task-dependent.

By randomizing subjects to either a day shift condition with normal alignment of the homeostatic and circadian processes, or a night shift condition with nearly opposite (i.e., more than 10.5 h shifted) alignment of the homeostatic and circadian processes, we created favorable empirical conditions [66,67] for dissociating the contributions of these two biological processes to neurobehavioral functioning as measured in the strictly controlled setting of the subsequent CR protocol. By also accounting for inter-individual differences in the statistical analysis [47], we further optimized our ability to isolate the circadian component of the temporal dynamics of neurobehavioral functioning [7]. Additionally, by selecting well-characterized [40,44,46] and functionally distinct [36,40] neurobehavioral assays, we avoided a range of measurement confounds encountered in the literature (e.g., practice effects, strategy changes, and dynamic speed/accuracy trade-offs) and enhanced our ability to detect task-dependent differences in circadian timing if such differences were present. Thus, our failure to detect differences in the circadian peaks of the three neurobehavioral assays is not an artifact of insufficient statistical power from inadequate sample size—although arguably a larger sample would have enhanced our ability to demonstrate even smaller differences—but rather indicates that with regard to timing, these assays were not affected differentially by endogenous circadian rhythmicity.

A limitation of our study is that the sample consisted of healthy young adults only and was predominately male. Age and sex differences in circadian rhythms have been reported [68,69], and whether those might interact with inter-task differences remains unknown. Importantly, another limitation is the small number of different assays we deployed. The imperative of repeated testing and the need for rest breaks to avoid cumulative time-on-task effects, as well as time windows needed for blood sampling and hourly snacks during the CR protocol, meant that only three assays could be included in the test battery.

Further research into task-dependence of the endogenous circadian impact on performance should incorporate other neurobehavioral functions, but selecting the right assays is not a straightforward matter. The so-called “task impurity problem” implies that any neurobehavioral task involves a number of interrelated cognitive processes that must be dissociable and distinguished to be able to interpret which, if any, of these processes are differentially affected by circadian rhythmicity [40,70]. Based on dissociations demonstrated in recent sleep deprivation studies, neurobehavioral (or cognitive) functions of particular interest for future studies may include attentional control [71], memory binding [72] and maintenance [73], and emotion regulation [74]. Referring to the sleep deprivation literature is relevant here, because our results indicate that the differences we observed in the overall temporal profiles of the KSS, DSST, and PVT with the circadian and homeostatic processes still intertwined (Figure 3) are attributable at least in part to differences between neurobehavioral functions in the influence of time awake through the homeostatic process. This more holistic perspective is consistent with a theoretical model in which the circadian process exerts global, top-down control over neurobehavioral functioning (placing peak circadian functioning in the “wake maintenance zone” as we have found); whereas the homeostatic process exerts local, bottom-up control based on the neuronal pathways involved, in a use-dependent manner that gives rise to differential degrees of impairment [38,42]. Our results add to a growing body of evidence consistent with that theoretical model [75,76,77].

Our findings have practical implications for shift-workers and others involved in around-the-clock operations [78,79,80]. If the circadian timing of neurobehavioral functioning were inherently task-dependent, this would present a formidable challenge in the prediction, assessment, and management of workplace risks for errors, incidents, and accidents [81,82]. However, while there are substantial inter-individual differences in the magnitude of neurobehavioral impairment to contend with in such settings [83], our results provide some reassurance that circadian peaks and troughs in subjective assessments of sleepiness are temporally aligned with those in objective performance deficits. Thus, biologically driven, circadian changes in neurobehavioral functioning should be predictable regardless of the tasks people perform—consistent with how it is implemented in current biomathematical models predicting neurobehavioral performance [12]. As such, there does not appear to be a fundamental need to adjust biomathematical model predictions based on what activities a person may be engaged in.

## 5. Conclusions

If there are inherent differences between tasks in the timing of the endogenous circadian peak, our carefully conducted study with comprehensive disentanglement of the circadian and homeostatic processes as well as the inter-individual differences therein would have been expected to manifestly expose them—but for the three distinct neurobehavioral tasks we investigated we found peak circadian performance clustering in the late biological afternoon (in the “wake maintenance zone”) during the CR protocol after both simulated day and night shift schedules. Our findings are in line with reports from previous CR studies [28,29] where, as Monk and colleagues observed: “between-task heterogeneity in circadian performance rhythms appeared to be absent when the sleep/wake cycle was suspended” [29] (p. 9). However, results from a forced desynchrony study [28], and from our systematic dissociation of the endogenous circadian process from the homeostatic process via juxtaposition of prior exposure to simulated day versus night shift conditions, suggest that between-task heterogeneity in circadian performance rhythms may be more broadly absent; and that any apparent circadian timing differences in other studies may actually be attributable to differential homeostatic effects and/or measurement confounds. From the available evidence, therefore, we parsimoniously conclude that circadian rhythmicity in neurobehavioral functioning is governed by a single endogenous circadian driver emanating from the central biological clock [84]. Further research with larger and more diverse samples and a wider spectrum of distinct neurobehavioral assays is needed to explore the limits of generalizability of this conclusion.

## Data Availability

Upon reasonable request from H.P.A.V.D. the data can be shared with researchers.
